# Improving image quality and diagnostic confidence for PRETEXT staging in pediatric hepatoblastoma using thin-slice and low-energy virtual monochromatic images in dual-energy CT with deep learning image reconstruction algorithm

**DOI:** 10.1186/s12880-026-02476-z

**Published:** 2026-05-30

**Authors:** Guangheng Yin, Tingting Guo, Jun Feng, Haoyan Li, Yaoyao Song, Yunxian Zhang, Yun Peng, Jihang Sun

**Affiliations:** 1 Department of Radiology, Beijing Children’s Hospital, Capital Medical University, National Center for Children’s Health, No.56, Nanlishi Road, Xicheng District, Beijing, 100045 China; 2 Department of Surgical Oncology, Beijing Children’s Hospital, Capital Medical University, National Center for Children’s Health, Beijing, 100045 China; 3 Medical Science Center, Yangtze University, No.1 Xueyuan road, Jingzhou, Hubei 434023 China

**Keywords:** Computed tomography angiography, Radiography, Dual-energy scanned projection, Hepatoblastoma, Deep learning, Image processing, Computer-assisted

## Abstract

**Background:**

Hepatoblastoma is the most common pediatric hepatic tumor, for which surgery is the primary treatment option. The PRETEXT (Pretreatment Extent of Disease) staging system, based on CT images, is a crucial basis for surgical planning. Therefore, improving the image quality and diagnostic confidence of PRETEXT staging impacts the overall therapeutic outcomes in pediatric hepatoblastoma.

**Objective:**

To investigate whether thin-slice 40 keV dual-energy CT (DECT) images combined with a deep learning image reconstruction (DLIR) algorithm can improve image quality and diagnostic confidence for the evaluation of PRETEXT staging for pediatric hepatoblastoma.

**Methods:**

This single-center retrospective study included 53 pediatric patients (mean age, 3.54 ± 2.26 years) with pathologically confirmed hepatoblastoma who underwent contrast-enhanced abdominal DECT. From the raw data, three distinct image series were reconstructed with a slice thickness of 0.625 mm for comparison: (A) standard-energy 68 keV VMI (virtual monoenergetic image) with 50% adaptive statistical iterative reconstruction-V (ASIR-V50%); (B) 68 keV VMI with high-level DLIR (DLIR-H); and (C) low-energy 40 keV VMI with DLIR-H. Objective image quality was quantified by the contrast-to-noise ratio (CNR) and Edge Rise Slope (ERS) of hepatic veins. Two independent radiologists performed PRETEXT staging and subjectively assessed image noise, hepatic vein visualization, and diagnostic confidence using a 5-point Likert scale.

**Results:**

The final PRETEXT staging results showed no statistically significant difference among the three image groups. However, objectively, the 40 keV DLIR-H images demonstrated significantly superior ERS (83.73 ± 46.50), indicating the sharpest vessel boundaries (*p* < 0.001), and CNR values for the hepatic veins. Subjectively, the 40 keV DLIR-H images received the highest scores for hepatic vein visualization and diagnostic confidence (*p* < 0.001), and was the only group consistently deemed sufficient to meet all diagnostic requirements for staging.

**Conclusion:**

The 0.625 mm thin-slice 40 keV VMI in DECT combined with DLIR-H reconstruction provides superior image quality and significantly enhances the diagnostic confidence for PRETEXT staging, and may be considered for routine clinical use.

## Background

Hepatoblastoma (HB) is the most common primary childhood malignant hepatic tumor, affecting approximately 1.5 per million children annually, typically under four years of age [1]. The cornerstone of curative therapy is complete surgical resection, often combined with neoadjuvant or adjuvant chemotherapy, which has elevated 5-year survival rates to over 80% [2, 3]. However, the onset of HB is insidious, often presenting with a large abdominal mass as the initial symptom. At the time of presentation, the tumor frequently compresses intrahepatic vessels and surrounding organs, which increases the difficulty of assessment and surgical treatment. Therefore, accurately performing preoperative clinical staging of HB to guide the selection of surgical approaches and the formulation of chemotherapy regimens is of significant importance.

**Fig. 1:**
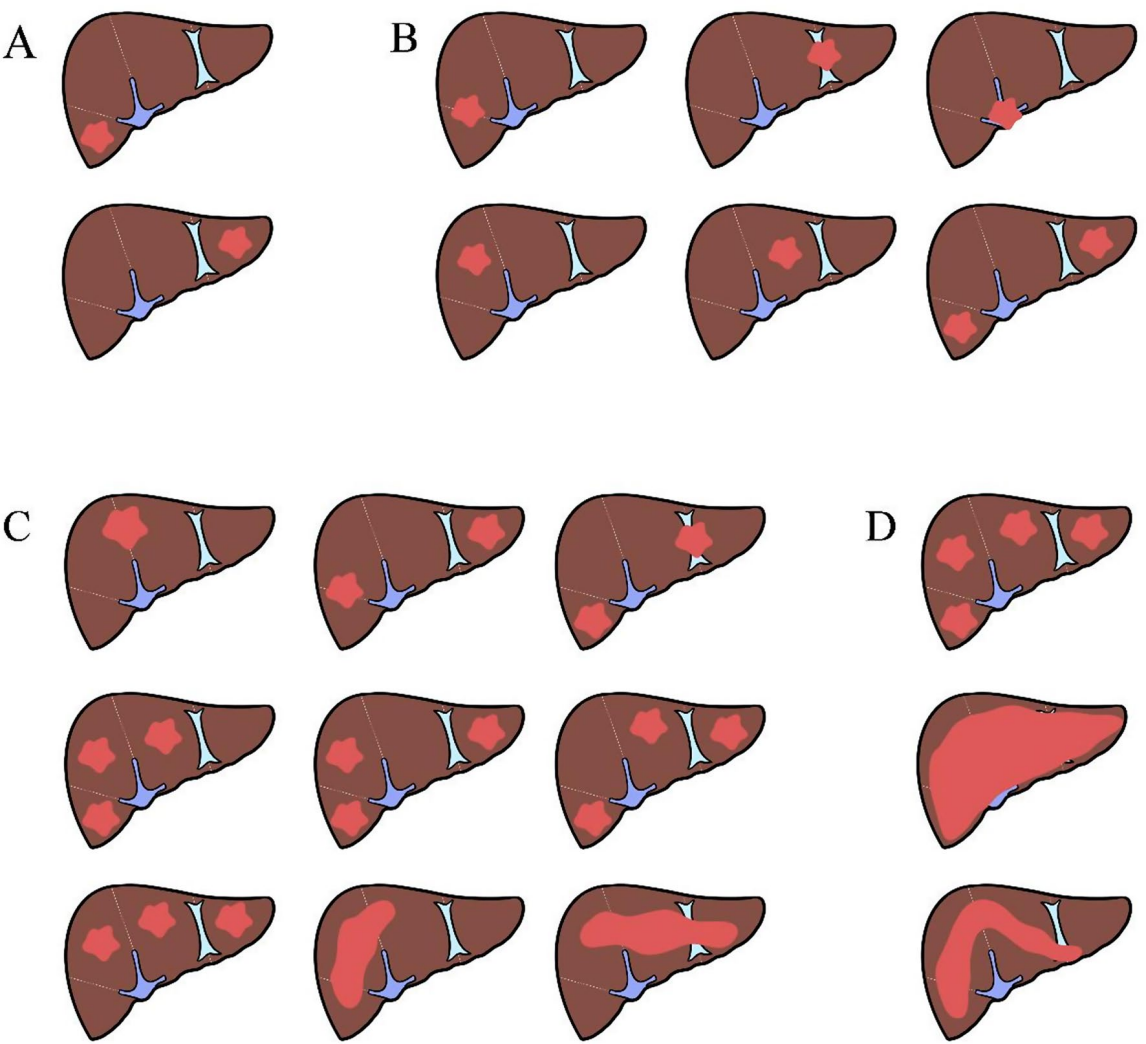
Schematic diagram of PRETEXT staging. **A** shows PRETEXT stage I: the tumor is confined to one liver section, with the other three contiguous sections free of tumor. **B** shows PRETEXT stage II: the tumor involves one or two liver sections, with two contiguous sections free of tumor. **C** shows PRETEXT stage III: two or three liver sections are involved, with one contiguous section remaining free of tumor. **D** shows PRETEXT stage IV: the tumor involves all four liver sections. Note: These four sections represent the simplified liver division for the PRETEXT system and should not be confused with the more detailed Couinaud segmental anatomy

The staging of hepatoblastoma primarily relies on the Pretreatment Extent of Disease (PRETEXT) staging system, developed by the International Society of Paediatric Oncology based on imaging examinations for HB. This system divides the liver into four sections and classifies tumors into stages I to IV based on the number and location of the affected sections (Fig. 1). PRETEXT staging enables preoperative risk stratification, aids in predicting surgical outcomes and long-term prognosis, and provides a basis for determining clinical treatment plans [4, 5], so improving the image quality of contrast-enhanced CT is crucial for accurate PRETEXT staging.

Dual-energy CT (DECT), by acquiring data at two different energy spectra during a single scan, provides additional diagnostic parameters beyond those of conventional CT images [6, 7] and improves the accuracy of artificial intelligence diagnostic models [8, 9]. Furthermore, the low-keV images in DECT enhance the attenuation of vessels in contrast-enhanced CT scans, thereby improving their visualization in CT angiography [10, 11], However, due to the lower penetration power of low energy photons, the low-keV images also significantly increase image noise [12]. In addition, in the preoperative assessment of pediatric hepatoblastoma, the hepatic veins are slender and often compressed by the tumor. The noise interference in low-keV images can easily lead to blurred vessel boundaries, further increasing the difficulty of PRETEXT staging. Consequently, its application value in the assessment of venous vessels required further validation.

Image noise may be reduced with the use of advanced reconstruction algorithms. The Deep Learning Image Reconstruction (DLIR) algorithm is developed to intelligently reduce image noise while maintaining natural image textures. It is based on a convolutional neural network (CNN) architecture and is trained by matching with high-quality phantom and actual clinical images reconstructed using Filtered Back Projection (FBP) reconstruction algorithm [13]. Compared to Adaptive Statistical Iterative Reconstruction-Veo (ASIR-V), it achieves a better balance between “noise reduction” and “detail preservation” [14, 15]. Therefore, utilizing the DLIR algorithm to optimize low-keV DECT images is expected to offset the noise interference associated with low-keV imaging while preserving its high-contrast advantage, thereby providing support for the detailed visualization of hepatic veins and PRETEXT staging. The 40 keV VMI with its energy level closest to the K-edge of iodine in DECT, provides the maximum theoretical contrast enhancement for vascular structures [16]. To this end, this study combines the use of DLIR and 40 keV DECT images to investigate whether this approach could improve image quality by maximizing contrast-to-noise ratio of vascular structures and hepatic lesions and subsequently improve diagnostic confidence and accuracy of PRETEXT staging for pediatric hepatoblastoma.

## Methods

### Design, setting, and participants

This study consecutively included the imaging data of pediatric patients with hepatoblastoma who underwent contrast-enhanced abdominal DECT scans for preoperative suspicion of hepatoblastoma from December 2021 to August 2024. The other inclusion criteria were: (I) a final diagnosis of hepatoblastoma was confirmed by postoperative pathology; and (II) age under 18 years. The exclusion criterion was incomplete clinical data or DECT imaging data. In total, 53 patients were included in the study, including 26 males and 27 females with median age of 3 years (range, 1.2 months to 10 years). To validate the accuracy of the radiological staging, the PRETEXT assessments were retrospectively correlated with intraoperative surgical findings, which served as the reference standard for anatomical involvement.

### Imaging equipment and protocol

DECT scans were performed on a 256 row CT scanner (Revolution CT, GE HealthCare, WI, USA) with rapid tube voltage switching between 80 and 120 kVp. A fixed tube current was selected based on the child’s body weight (200 mA for 0–15.9 kg; 240 mA for 16–32 kg). Other parameters were: detector width of 40 mm, rotation time of 0.5 s, pitch of 1.375:1, and a scan field of view of 35 cm. The contrast agent injection protocol involved a dose of 1.6 ml/kg (for 0–15.9 kg) or 1.4 ml/kg (for 16–32 kg) based on the child’s body weight, administered via a single-head power injector. The injection duration was 17 s (for 0–15.9 kg) or 21 s (for 16–32 kg). The contrast-enhanced DECT scan was acquired 55 s after the initiation of contrast injection. All scans were performed while the children were in a quiet state. If a child was crying and uncooperative, 10% chloral hydrate was administered at a dose of 0.4 ml/kg, and the scan was performed after the child was in a quiet sleep.

The acquired raw data were used to reconstruct 68 keV images with 50% ASIR-V and DLIR with high setting (DLIR-H), all images were reconstructed using a standard reconstruction kernel. Concurrently, a set of 40 keV images was reconstructed using DLIR-H. The reconstruction matrix was 512 × 512 with slice thickness and slice interval of 0.625 mm for all images.

### Outcome measurement

The primary endpoint of this study was the subjective image quality assessment and diagnostic confidence for PRETEXT staging. Objective image quality metrics (CNR and ERS) were measured as part of image quality assessment and the final PRETEXT staging agreement were evaluated as secondary endpoints.

#### Qualitative image quality evaluation

All images were transferred to a GE AW4.7 workstation, where patient and examination information as well as specific reconstruction types, were anonymized to ensure the readers were fully blinded. Two radiologists, with 19 and 5 years of experience in pediatric tumor assessment, independently performed the PRETEXT staging. To reduce recall bias, all image series from different reconstruction methods were anonymized, separated, and evaluated in randomized order. The staging assessment was conducted according to the criteria of the International Childhood Hepatic Tumour Strategy Group (SIOPEL)[5]. Concurrently, they independently evaluated the image quality using a 5-point scale (where 3 = acceptable and 5 = excellent). The evaluation assessed overall image noise, visualization of the hepatic veins, and confidence in PRETEXT staging. The detailed scoring criteria are presented in Table 1. During the evaluation process, both reviewers were free to adjust the window width and window level and were permitted to use multiplanar reformation (MPR) and three-dimensional (3D) reconstruction tools to aid in their observation.

**Table 1:** Table of scoring criteria for image quality

	5 points	4 points	3 points	2 points	1 point
Overall Image Noise	Minimal noise, does not affect diagnosis	Low noise, does not affect diagnosis	Moderate noise, sufficient for diagnosis	High noise, limits diagnostic confidence	Severe noise, precludes diagnosis
Vessel Visualization (Hepatic Veins)	Sharply defined borders	Clearly visible borders	Adequately visible for staging	Blurred / partially obscured borders	Indistinguishable / not visible
Diagnostic Confidence (PRETEXT Staging)	High confidence	Good confidence	Sufficient confidence, with some uncertainty	Low confidence	No confidence

#### Quantitative image quality measurement

Upon completion of the subjective evaluation, the two radiologists jointly used measurement tools on the workstation to objectively measure the CT attenuation values and standard deviations (SD). To minimize consensus bias and technical measurement variability in small pediatric vessels, the two radiologists jointly determined the locations for the regions of interest (ROIs), specifically selecting subjectively uniform areas within the vessel lumen to avoid vessel walls and partial-volume effects. If there was a disagreement regarding the measurement location, a third, senior radiologist was consulted to determine the final ROI placement. They then calculated the contrast-to-noise ratio (CNR) of the hepatic veins (Formula 1).

1 $$\>CNR = {{\left( {{\rm{C}}{{\rm{T}}_{{\rm{HV}}}} - {\rm{C}}{{\rm{T}}_{{\rm{Hepatic}}}}} \right)} \over {{1 \over 2} \times \>\left( {S{D_{HV}} + S{D_{Liver}}} \right)}} \times \>100\% $$

Where, CT_HV_ and CT_Hepatic_ represent the CT attenuation values of the hepatic vein and the hepatic parenchyma, respectively, and SD_HV_ and SD_Hepatic_ represent the SD values of the hepatic vein and the hepatic parenchyma, respectively.

Subsequently, the edge rise slope (ERS) at the interface between the hepatic vein and hepatic parenchyma was measured. To avoid inaccuracies caused by motion or severe partial-volume effects, the radiologists carefully selected vessel segments with clear edges and no obvious motion artifacts for ERS measurement. First, a line profile was drawn across the junction between the hepatic vein and the hepatic parenchyma to record the CT attenuation values (HU) along the line. The edge rise distance (ERD) was then calculated as the difference between the x-coordinates at which the attenuation value reached 90% (X1) and 10% (X2) of its maximum value (ERD = X1 - X2). The ERS was then calculated according to Formula 2.

2 $$\>{\rm{ERS}} = {{\left( {{\rm{H}}{{\rm{U}}_{90\% }} - {\rm{H}}{{\rm{U}}_{10\% }}} \right)} \over {{\rm{ERD}}}}$$

**Fig. 2:**
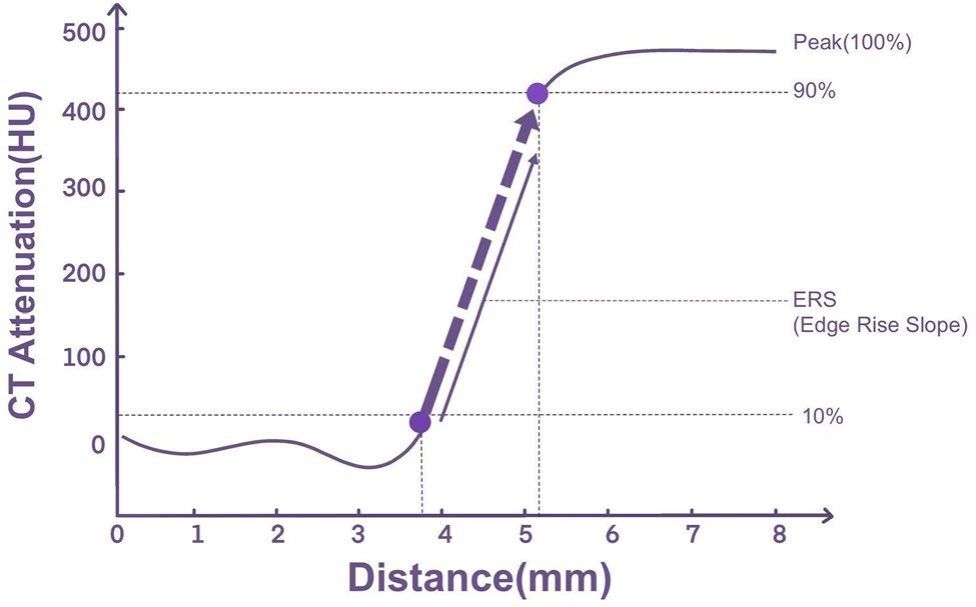
Schematic diagram illustrating the calculation of the edge rise slope (ERS)

Herein, HU_90%_ represents 90% of the maximum CT attenuation value, and HU_10%_ represents 10% of the maximum CT attenuation value. The methodology for ERS calculation is illustrated in Fig. 2.

### Statistical analyses

All statistical analyses were performed using SPSS software (version 26.0). Subjective evaluation results were described using the percentage of each score and percentiles (P25, P50, P75), and the Friedman test was employed for analysis. Objective evaluation results were presented as mean ± standard deviation. If the data met the assumptions of normal distribution and homogeneity of variance, one-way ANOVA was used for statistical analysis. When a statistically significant difference was identified, the Bonferroni post hoc test was conducted for pairwise comparisons. A non-parametric test was used when the data did not conform to a normal distribution. A P-value of < 0.05 was considered to be statistically significant. The kappa statistic was used to compare the inter-rater agreement of the subjective scores between the two physicians. Two experienced radiologists independently performed the subjective image quality scoring for all image sets. For the statistical analysis of subjective outcomes, the ratings from both readers were included jointly as individual observations and were not averaged into a single composite score. The kappa statistic was used to assess the inter-rater agreement of subjective scores between the two radiologists, with the agreement interpreted as: < 0.40 (poor), 0.40–0.59 (fair), 0.60–0.74 (good), and ≥ 0.75 (excellent). All subjective scores from both radiologists were included in the subsequent statistical analyses as the final scores for the subjective evaluation of image quality, and no data were excluded or merged based on the inter-rater agreement results. The Friedman test was employed to compare the three groups. When a statistically significant difference was identified, post-hoc pairwise comparisons were performed using the Wilcoxon signed-rank test with a Bonferroni correction.

Given the rarity of pediatric hepatoblastoma, the sample size was limited by the availability of consecutive eligible cases. A post-hoc power analysis was performed for the primary endpoint.

## Results

### Patient characteristics

A total of 53 consecutive patients who underwent an abdominal contrast-enhanced DECT scan and were definitively diagnosed with hepatoblastoma were retrospectively selected. The mean age was 3.54 ± 2.26 years, and the male-to-female ratio was 26:27. Correlation with surgical findings confirmed the accuracy of the final PRETEXT staging in all patients across all three image groups, with the surgical resection findings being consistent with the CT-based assessment of tumor extent and anatomical involvement. The detailed radiation dose metrics, including CTDIvol, DLP for the CT examinations, are summarized in Table 2.

**Table 2:** Demographic, clinical and technical characteristics of the study population

Characteristics	Value
**Age (years)**	
Mean ± SD	3.54 ± 2.26
Range	0.30–11.00
**Sex**,** n (%)**	
Male	26(49.06%)
Female	27(50.94%)
**Body Weight (kg)**	
Mean ± SD	16.60 ± 6.90
Range	5.00–37.00
**PRETEXT Stage**,** n (%)**	
Stage I	13 (24.52%)
Stage II	7 (13.21%)
Stage III	11 (20.75%)
Stage IV	22 (41.51%)
**CTDIvol (mGy)**	
Mean ± SD	3.80 ± 0.39
Range	3.49–4.28
**DLP (mGy·cm)**	
Mean ± SD	96.35 ± 25.67
Range	61.27–188.11
**Injection Rate (mL/s)**	
Mean ± SD	1.30 ± 0.39
Range	0.40–2.50
**Contrast Volume (mL)**	
Mean ± SD	24.45 ± 8.89
Range	8.00–52.00

### Subjective scoring

**Fig. 3:**
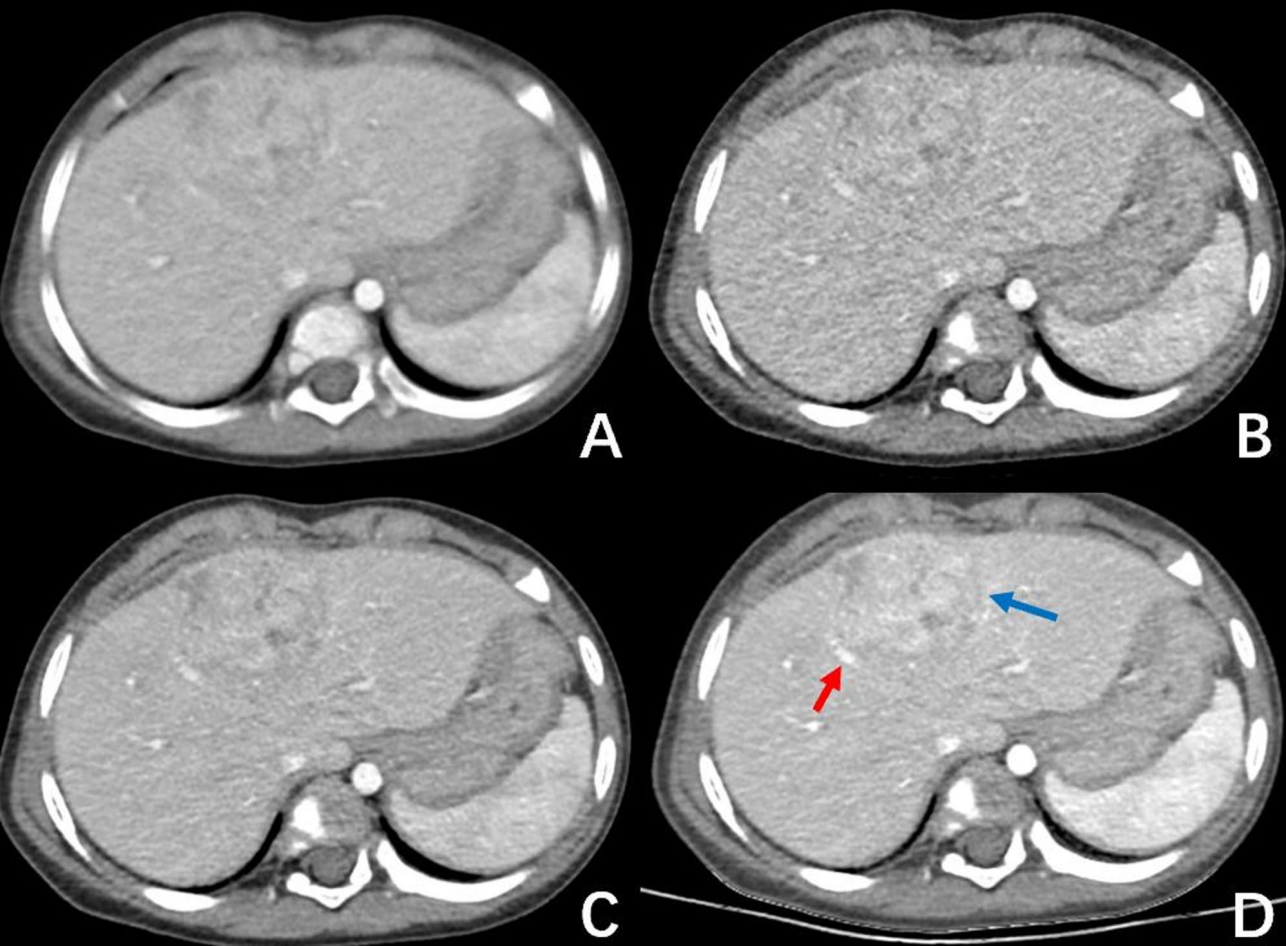
Dual-energy CT (DECT) scan images of a hepatoblastoma in a pediatric patient. **A**: 68 keV, 5 mm, 50% Adaptive Statistical Iterative Reconstruction-V (ASIR-V). This thick-slice image was used as a standard-care reference image. It exhibits the least noise, but the thick slice image results in blurred vascular margins, leading to poor visualization of the middle hepatic vein and low staging confidence. **B**: 68 keV, 0.625 mm, 50% ASIR-V. The hepatic veins are visible with a medium level of image noise; however, the demarcation of the tumor mass is poor, resulting in low staging confidence. **C**: 68 keV, 0.625 mm, Deep Learning Image Reconstruction with high setting (DLIR-H). The noise level is low, with adequate visualization of the hepatic vein; the tumor is more clearly demonstrated, yielding improved staging confidence. **D**: 40 keV, 0.625 mm, DLIR-H. The image has low noise; the middle hepatic vein (red arrow) shows high contrast and is clearly visualized. The tumor was determined to be located between the middle hepatic vein and the hepatic fissure, abutting the middle hepatic vein, and was classified as Pretreatment Extent of Disease (PRETEXT) stage II with high diagnostic confidence. The image shows the sharpest demarcation between the tumor margin and the liver parenchyma (blue arrow), allowing better determination of the precise extent of the tumor

**Fig. 4:**
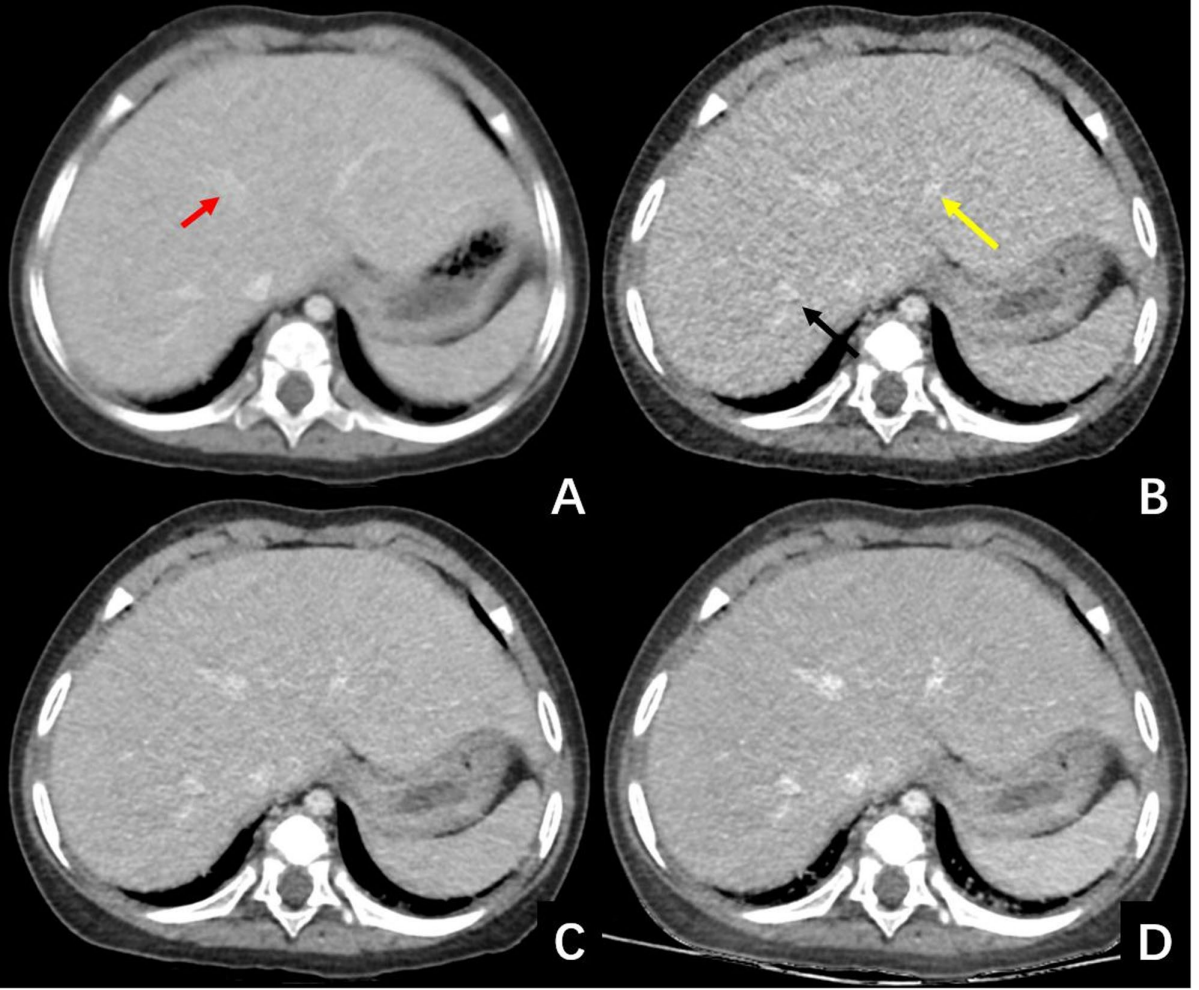
Dual-energy CT (DECT) images of a hepatoblastoma in a pediatric patient. **A**: 68 keV, 5 mm, 50% Adaptive Statistical Iterative Reconstruction-V (ASIR-V). This thick-slice image was used as a standard-care reference image. The image noise is relatively low; however, due to the partial volume effect of the 5 mm slice thickness, the middle hepatic vein (red arrow) appears blurred and discontinuous. **B**: 68 keV, 0.625 mm, 50% ASIR-V. The thin-slice image improves visualization of the middle hepatic vein, but also leads to increased image noise. Consequently, the left hepatic vein is poorly visualized (yellow arrow), and the borders of the right hepatic vein are blurred (black arrow). Nevertheless, continuous observation through the thin slices allows assessment of the relationship between the vessels and the tumor mass. **C**: 68 keV, 0.625 mm, Deep Learning Image Reconstruction with high setting (DLIR-H). The use of DLIR-H reduces image noise and improves the sharpness of the right hepatic vein margins, but it does not resolve the poor visualization of the left hepatic vein. **D** 40 keV, 0.625 mm, DLIR-H. At 40 keV, the noise is acceptable, and the contrast of the intrahepatic veins is significantly enhanced, allowing clear visualization of all intrahepatic veins

**Fig. 5:**
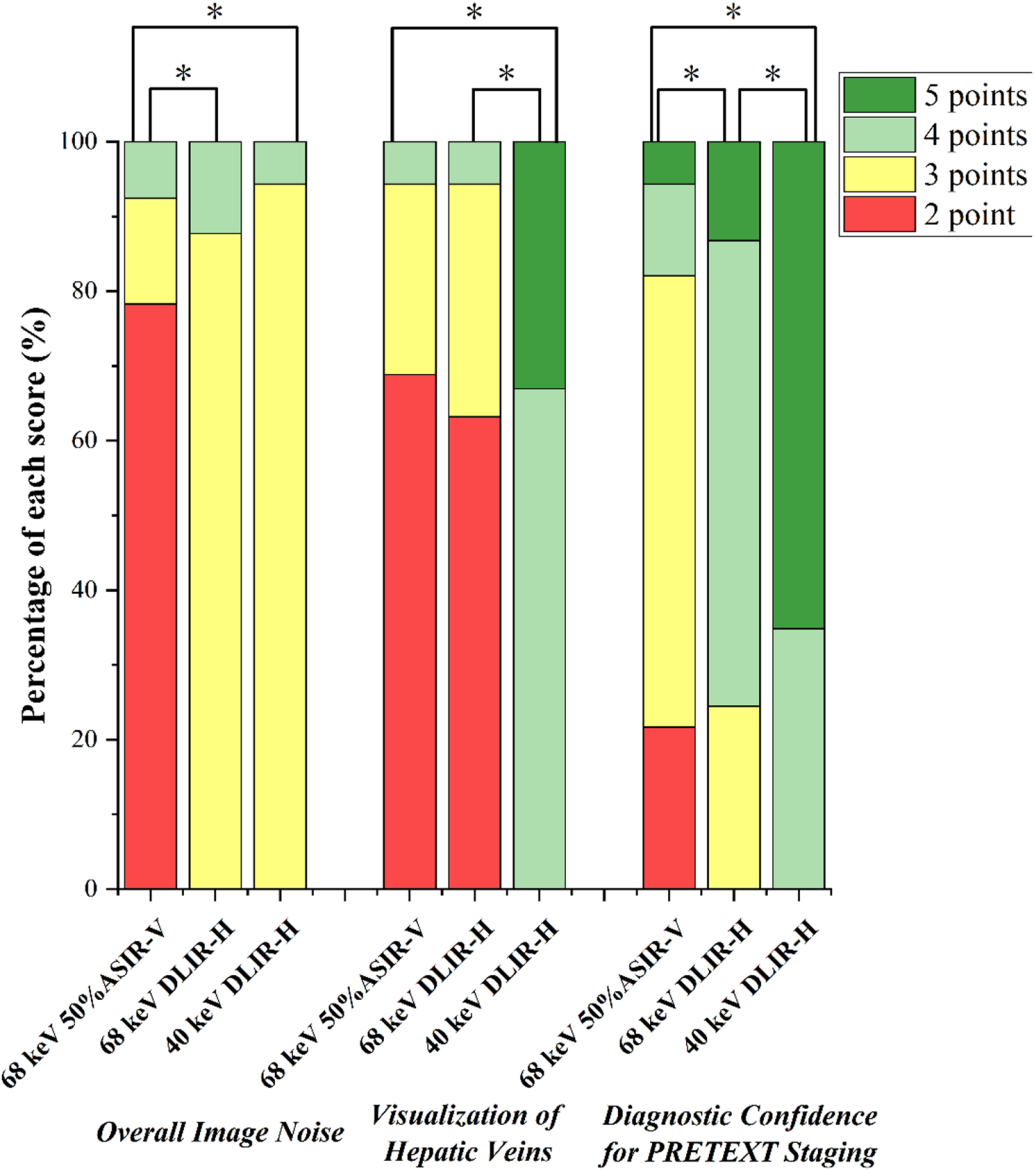
A percentage stacked bar chart showing the distribution of subjective scores in overall image noise, visualization of hepatic veins, and diagnostic confidence for PRETEXT staging for the three groups, include combined scores from two readers. As indicated by the legend, red corresponds to a score of 2, yellow to 3, light green to 4, and dark green to 5. The 68 keV 50%ASIR-V consists of images reconstructed with 68 keV, 0.625 mm, and 50% ASIR-V (Adaptive Statistical Iterative Reconstruction); The 68 keV DLIR-H with 68 keV, 0.625 mm, and DLIR-H (Deep Learning Image Reconstruction); and the 40 keV DLIR-H with 40 keV, 0.625 mm, and DLIR-H. An asterisk (*) indicates a significant difference between two groups (p < 0.001). The results demonstrated that there was no significant difference in image noise scores between the 68 keV 50%ASIR-V and the 68 keV DLIR-H. For both vessel contrast and staging diagnostic confidence, the 40 keV DLIR-H achieved the highest scores, which were significantly different from the other two groups

**Fig. 6:**
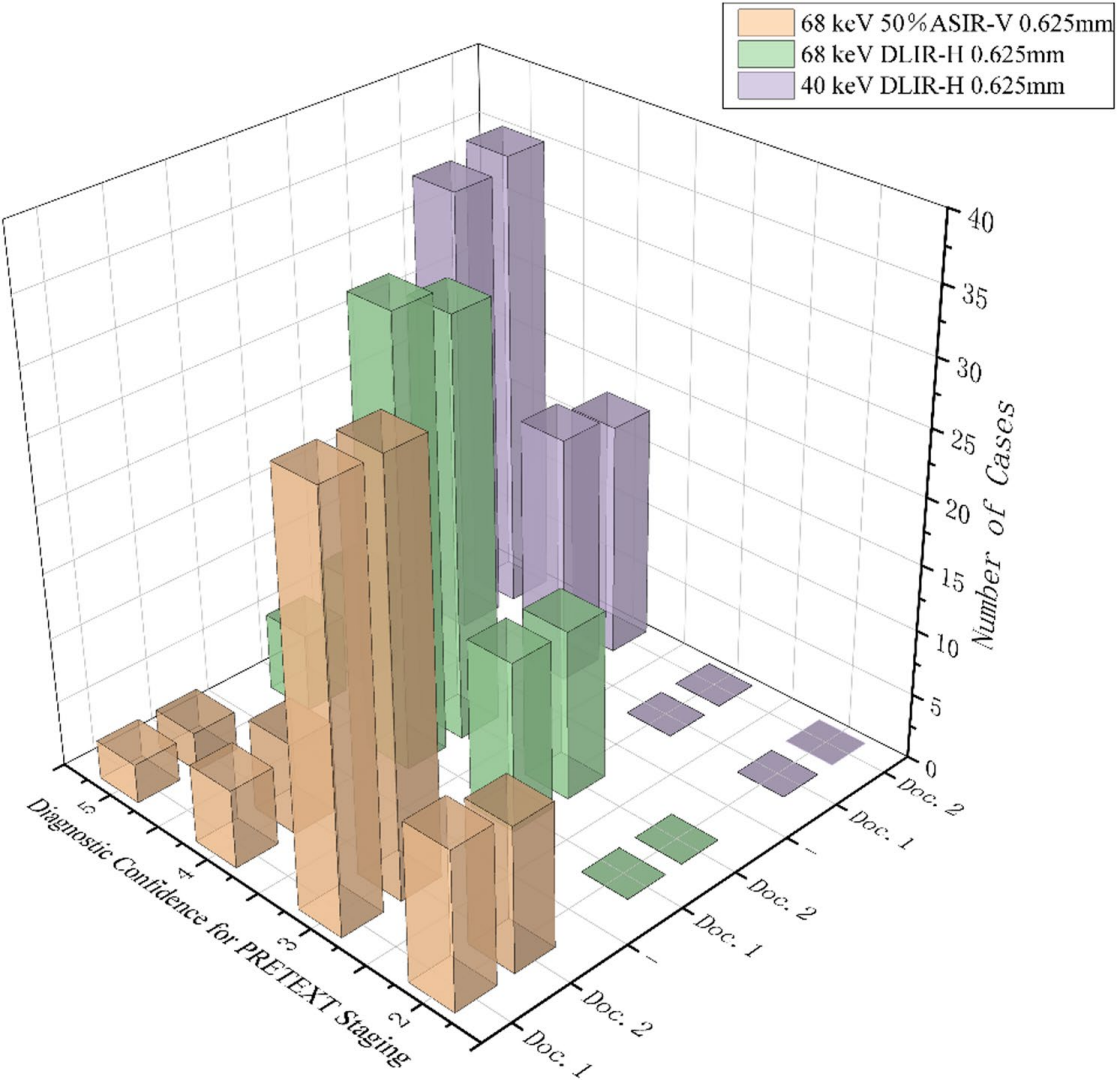
A bar chart showing the statistics on diagnostic confidence for PRETEXT staging from two physicians for the three image groups. The orange bars represent the 68 keV, 0.625 mm, 50% ASIR-V (Adaptive Statistical Iterative Reconstruction-V) images; the green bars represent the 68 keV, 0.625 mm, Deep Learning Image Reconstruction with high setting (DLIR-H) images; the purple bars represent the 40 keV, 0.625 mm, DLIR-H images. Doc.1 was a physician with 19 years of diagnostic experience, and Doc.2 was a physician with 5 years of diagnostic experience. The evaluations of the two physicians showed good agreement

The PRETEXT staging results assessed by two radiologists across the three image sets achieved 100% agreement (Kappa = 1.0). Regarding the overall image noise scores, 68 keV DLIR-H and 40 keV DLIR-H had the same median score, with no significant difference between their results (*p* = 0.47, *r* = 0.06, negligible effect), both met the diagnostic requirements and were superior to 68 keV 50%ASIR-V (*p* < 0.001, *r* = 0.67–0.73, large effect). For the vessel contrast score, 40 keV DLIR-H had the highest values, with its median score being significantly higher than those of both 68 keV 50%ASIR-V and 68 keV DLIR-H (*p* < 0.001, *r* = 0.86–0.89, large effect) (Fig. 3); the corresponding quartiles of 68 keV 50%ASIR-V and 68 keV DLIR-H were identical, and their median (Q2) did not meet the diagnostic requirements (Fig. 4). In terms of diagnostic confidence for PRETEXT staging, the 40 keV DLIR-H group showed the highest scores, with a median of 5.0 and significant differences in all pairwise comparisons with 68 keV 50% ASIR-V and 68 keV DLIR-H groups (*p* < 0.01, *r* = 0.51–0.98, large effect). The proportions of confident ratings (scores 4–5) were 18.0% for 68 keV 50% ASIR-V, 75.5% for 68 keV DLIR-H, and 100% for 40 keV DLIR-H. Detailed results are shown in Table 3; Fig. 5. There was good inter-rater agreement between the two evaluating physicians (Fig. 6), with a kappa value of 0.97 (*p* < 0.05). Furthermore, we evaluated PRETEXT annotation factors beyond basic staging, such as lesion attenuation, enhancement pattern, and morphological characteristics. Comparative assessment demonstrated that there were no statistically significant differences in the diagnostic results between image sets with different reconstruction types.

### Objective measurement results

**Fig. 7:**
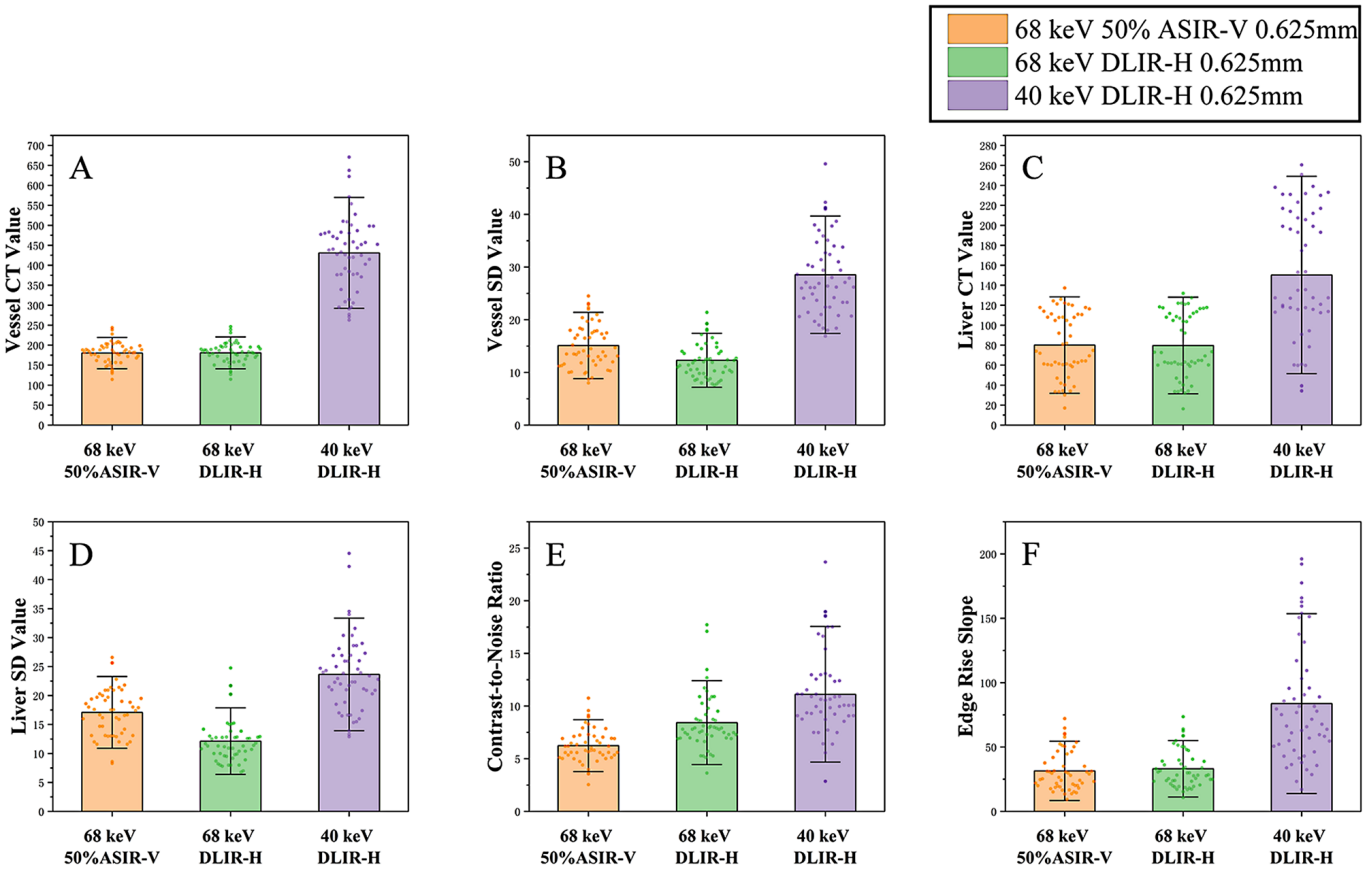
As shown in the figure, the orange bars represent the 68 keV, 0.6 mm, 50% ASIR-V (Adaptive Statistical Iterative Reconstruction-V) images; the green bars represent the 68 keV, 0.6 mm, Deep Learning Image Reconstruction with high setting (DLIR-H) images; and the purple bars represent the 40 keV, 0.6 mm, DLIR-H images. The graphs show: CT attenuation value for the vessel (**A**), liver (**C**); SD value for the vessel (**B**), liver (**D**); **E** shows the contrast-to-noise ratio (CNR); **F** shows the edge rise slope (ERS). The graphs indicate that the CT attenuation value of the 40 keV DLIR-H is significantly higher than those of the other groups, with no significant difference in CT attenuation values between the other two groups. In terms of noise, the 40 keV DLIR-H is higher than the other groups

The measurement results showed that 40 keV DLIR-H had the highest hepatic vein CT attenuation values at 430.97 ± 92.56 HU, and highest hepatic tumor CT attenuation values at 150.21 ± 65.86 HU among the three image groups which were all significantly higher than 68 keV 50%ASIR-V and 68 keV DLIR-H (*p* < 0.05); while there were no statistically significant differences between 68 keV 50%ASIR-V and 68 keV DLIR-H. Regarding image noise for hepatic vessels and the hepatic tumor, 68 KeV DLIR-H had the lowest noise, while 40 keV DLIR-H had the highest (*p* < 0.05). The CNR measurement results indicated that 40 keV DLIR-H had the highest CNR, which was significantly higher than the other two groups (*p* < 0.05). The ERS measurement results showed that 40 keV DLIR-H was the highest at 83.73 ± 46.54 (*p* < 0.05), indicating better vessel edge sharpness and spatial resolution, as well as the clearest vessel depiction, with statistically significant differences found in comparison with the other two groups. Detailed results are shown in Table 4; Fig. 7.

## Discussion

DECT has been widely adopted in adult clinical practice, and its application in pediatric radiology is also rapidly expanding [17]. In contrast-enhanced CT (CECT) examinations, in particular, the advantage of low-keV imaging in boosting the CT attenuation values of iodine is extensively utilized [18]. The PRETEXT staging of pediatric hepatoblastoma is crucial for determining treatment strategies, and its assessment heavily relies on the clear visualization of vascular structures on CECT. Therefore, low-keV virtual monoenergetic imaging (VMI) is an ideal choice for enhancing the quality of such images. However, the higher image noise inherent in low-keV images can impair the observation of fine details. Deep learning image reconstruction (DLIR) technology is promising for reducing noise while preserving image details, thereby optimizing overall image quality. This study demonstrated that the combination of DLIR with 40 keV images can enhance vascular contrast while maintaining image noise at diagnostically acceptable levels, thus improving image quality and reducing low or uncertain diagnostic confidence ratings (scores 1–3) for PRETEXT staging from 82.1% with 68 keV 50%ASIR-V and 24.5% with 68 keV DLIR-H to 0% with 40 keV DLIR-H.

Previous studies have shown that 3–5 mm thick-slice images result in fewer total images and lower noise, enabling good visualization of structures [19]. However, the partial volume effect inherent in thick-slice images often leads to blurring of the margins of small vessels (e.g., hepatic vein branches), failing to meet assessment requirements [20]. Consequently, thin-slice images (0.625–1 mm) are necessary to supplement the observation of fine anatomical details. Under identical scanning conditions, although 0.625 mm images offer higher spatial resolution, which is advantageous for visualizing minute vessels, their intrinsically higher noise level reduces the contrast-to-noise ratio (CNR). In our study, the 0.625 mm thin-slice images, when used alone, failed to meet diagnostic standards in terms of both noise level and subjective scores for vascular contrast, thus being insufficient for overall diagnostic needs. Therefore, in clinical practice, a combination of both image types (thin slice and thick slice images) is typically required for observation, representing a compromise between image noise and anatomical detail. However, this approach inevitably consumes more time and may introduce potential risks into the diagnostic process. Our findings suggest that the use of 40 keV DLIR-H images may reduce this dependence by providing acceptable noise and clearer vascular delineation in a single thin-slice dataset; nevertheless, because workflow-related metrics were not directly assessed in this study, this advantage should be regarded as potential rather than proven.

Previous research has established that advanced reconstruction methods like DLIR can significantly reduce image noise [21], and multiple studies have previously demonstrated the superiority of DLIR over Adaptive Statistical Iterative Reconstruction (ASIR) in terms of image quality at the same slice thickness [22–26]. Our study showed that at the same acquisition energy (68 keV 50%ASIR-V vs. 68 keV DLIR-H), high-strength DLIR (DLIR-H) achieved a significantly lower noise than the conventional high-strength ASIR-V while maintaining spatial resolution, thereby improving subjective diagnostic confidence and objective metrics such as the edge rise slope (ERS) for the 0.625 mm images, leading to a substantial improvement in image quality. This finding is consistent with the conclusions of Oostveen et al. [27]. Many of the DLIR-related application has been primarily focused on CT angiography (CTA) examinations [28–30], our study extended its application to the parenchymal phase of contrast-enhanced CT.

Our results showed that while combining DLIR with thin-slice images reduces noise, it did not change the CT attenuation values of vessels to significantly enhance the subjective scores for vascular contrast (68 keV 50%ASIR-V vs. 68 keV DLIR-H). Therefore, at a conventional 68 keV energy level, a single-phase thin-slice image optimized solely by DLIR was insufficient to fully meet the assessment demands for PRETEXT staging in contrast enhancement. On the other hand, the low-keV VMI technique in DECT can significantly enhance vascular contrast. To this end, our study utilized 40 keV VMI images, and our results demonstrated an 114.16% increase in vascular CT attenuation values compared to the 68 keV images, leading to a significant improvement in subjective scores for vascular contrast. Combined with the high spatial resolution of 0.625 mm thin slices and DLIR-H, this technique markedly enhanced the conspicuity of small vessels and diagnostic confidence for PRETEXT staging. Previous studies have shown that low-keV images, such as those at 40 keV, introduce additional image noise [12, 31, 32]. Conventional iterative reconstruction methods, including ASIR-V, may be insufficient to suppress this amplified noise without compromising image texture, which has limited the routine diagnostic use of standard 40 keV reconstructions in clinical practice [33, 34]. In this context, the favorable performance of 40 keV DLIR-H in our study likely reflects the combined advantage of low-keV contrast enhancement and DLIR-based noise reduction. Nevertheless, because a 40 keV conventional iterative reconstruction group was not included, the individual contributions of the low energy setting and the reconstruction algorithm could not be fully differentiated. Our objective measurements confirmed this, showing that even with DLIR-H, the noise in 40 keV images was the highest among the three groups but with more natural appearance, which aligns with theoretical expectations. Crucially, however, due to the K-edge effect of iodine contrast agent at 40 keV, the increase in vascular CT attenuation values far surpassed the increase in noise. This resulted in the highest hepatic vein CNR among the three groups, along with a significant improvement in ERS, indicating sharper and more distinct vessel margins. This is crucial for accurately delineating the course and interfaces of small vessels and the anatomical relationship between the tumor and vasculature, effectively reducing artifacts and blurring caused by the partial volume effect [32]. Furthermore, the more natural noise appearance and high CNR mitigated the impact of objective noise on subjective perception, allowing the subjective noise scores to meet diagnostic requirements. Recent studies in adult liver CT have demonstrated that the combination of low-keV VMI and DLIR significantly improves the conspicuity of hypervascular liver lesions and precise vascular mapping [35, 36]. Our findings align with these adult studies and further confirmed that these technical advantages could effectively translate to pediatric oncology, which is particularly crucial given the smaller caliber of pediatric vessels. Furthermore, while this study primarily focused on hepatic vein visualization, it is important to note that PRETEXT staging also heavily relies on lesion conspicuity. The 40 keV VMI technique inherently boosts iodine attenuation due to the K-edge effect, which not only improves vascular contrast but also significantly enhances tumor-to-liver contrast. This increased lesion conspicuity allows for a much clearer delineation of tumor margins and its precise anatomical relationship with adjacent liver sections. Consequently, the 40 keV DLIR-H approach provides critical additional information that is highly relevant for accurate PRETEXT staging and subsequent surgical planning. In summary, analysis of these results indicated that the 40 keV images with DLIR-H were far superior to those of the other groups in terms of vascular contrast, diagnostic confidence for staging, and the objectively measured ERS. Our results demonstrated that the combination of DLIR-H with 40 keV, 0.625 mm imaging in DECT provided the most diagnostic information, with the best vascular contrast and spatial resolution, while maintaining noise within an acceptable range. This approach was markedly superior to the conventional 68 keV 0.625 mm imaging.

Interestingly, despite these significant improvements in image quality and reader confidence, the final PRETEXT staging results did not significantly differ among the reconstruction groups. The concordance between CT-based PRETEXT staging and surgical findings across all reconstruction groups supported the reliability of CT for preoperative staging in pediatric hepatoblastoma. However, these findings suggested that the main clinical value of 40 keV DLIR-H in the present cohort lied in improved diagnostic confidence and interpretability, while final PRETEXT staging remained unchanged across the three reconstruction groups. In pediatric radiology, adhering to the ALARA (As Low As Reasonably Achievable) principle and minimizing contrast load are top priorities. Compared with the published U.S. pediatric benchmark for contrast-enhanced abdomen/pelvis CT [37], our mean CTDIvol (3.80 ± 0.39 mGy) was slightly higher than the reported 2.9 mGy value, whereas our mean DLP (96.35 ± 25.67 mGy·cm) was close to the reported 100 mGy·cm value; however, this comparison should be interpreted cautiously because the benchmark was established using age- and size-based registry data. Although cases were not formally categorized as equivocal during image interpretation in our study, the lower confidence ratings in the standard reconstruction groups and the representative findings in Fig. 3 suggested that some examinations were visually more challenging because of blurred vessel margins or indistinct tumor–vessel interfaces. In such situations, the improved vascular contrast and sharper margins provided by 40 keV DLIR-H images demonstrated their ability to facilitate image interpretation and increase diagnostic confidence. These findings also suggested a potential opportunity for future protocol optimization; however, whether this technique can support reductions in radiation dose or contrast medium volume without compromising diagnostic performance requires prospective validation.

It is worth noting that while our objective measurements primarily focused on the hepatic veins, comprehensive surgical planning and accurate PRETEXT staging depend on a wider range of anatomical considerations. We specifically selected the hepatic veins for quantitative analysis because their enhancement density in the venous phase is similar to that of the portal vein, making them a reliable representative for evaluating the portal venous system. Furthermore, hepatic veins are inherently slender and highly susceptible to tumor compression, making them an ideal “stress test” for image optimization techniques. However, in routine clinical practice, evaluating the venous system alone is insufficient. A complete surgical assessment also necessitates evaluating the hepatic artery anatomy during the arterial phase, as well as determining inferior vena cava involvement, biliary anatomy, and the presence of extrahepatic disease. Future studies should extend the application of the 40 keV DLIR-H technique to these diverse anatomical parameters and multi-phase scans to meet comprehensive surgical needs.

This study also has certain limitations. First, the single-center, retrospective design and small sample size without age stratification, and the inclusion of only surgically treated, pathologically confirmed cases may introduce potential selection bias, as more complex or unresectable tumors may have been excluded. The limited sample size precluded a formal subgroup analysis to determine whether the benefit of improved image quality would be greater in more complex cases, such as tumors adjacent to major hepatic vessels or with subtle tumor–vessel interfaces. These situations may have been underrepresented in the present cohort, which could have reduced the opportunity to appreciate the potential value of improved image quality under more challenging staging conditions. Second, inconsistent sedation protocols may have caused subtle motion artifacts, potentially introducing variability in objective quality metrics like ERS. ROI placement for quantitative analysis was determined jointly by the two radiologists to improve measurement consistency; however, this approach may also have introduced a degree of consensus bias. Third, although surgical findings served as the reference standard, the study demonstrated improved diagnostic confidence rather than enhanced staging accuracy, as no discordant PRETEXT staging occurred among the image groups. Without a 40 keV conventional reconstruction comparator, the respective effects of low energy level and reconstruction algorithm could not be fully separated. Therefore, a larger study involving more equivocal cases is needed to determine whether the improved image quality translates to higher staging accuracy. Fourth, because the same readers evaluated multiple image reconstructions from the same patients, case recognition and recall bias could not be fully excluded despite the randomization procedure. Additionally, long-term postoperative outcomes were not evaluated. Finally, while our findings suggest a theoretical potential for reducing radiation and contrast doses, future prospective trials are essential to confirm these benefits in clinical practice.

**Table 3:** Comparison of subjective image scores

	68 keV 50%ASIR-V		68 keV DLIR-H		40 keV DLIR-H		F	p	p(68 keV 50%ASIR-Vvs.68 keV DLIR-H)	p(68 keV 50%ASIR-Vvs.40 keV DLIR-H)	p(68 keV DLIR-Hvs.40 keV DLIR-H)
	Quartiles	percentage	Quartiles	percentage	Quartiles	percentage					
Overall Image Noise	2.00(Q1)2.00(Q2)2.00(Q3)	2.00(78.3%);3.00(14.2%);4.00(7.5%)	3.00(Q1)3.00(Q2)3.00(Q3)	3.00(87.7%)4.00(12.3%)	3.00(Q1)3.00(Q2)3.00(Q3)	3.00(94.3%);4.00(5.7%)	156.02	<0.001	<0.001	<0.001	0.47
Visualization of Hepatic Veins	2.00(Q1)2.00(Q2)3.00(Q3)	2.00(68.9%);3.00(25.5%);4.00(5.7%)	2.00(Q1)2.00(Q2)3.00(Q3)	2.00(63.2%);3.00(31.1%);4.00(5.7%)	4.00(Q1)4.00(Q2)5.00(Q3)	4.00(67.0%);5.00(33.0%)	203.28	<0.001	0.68	<0.001	<0.001
Diagnostic Confidence for PRETEXT Staging	3.00(Q1)3.00(Q2)3.00(Q3)	2.00(21.7%); 3.00(60.4%); 4.00(12.3%);5.00(5.7%)	3.75(Q1)4.00(Q2)4.00(Q3)	3.00(24.5%); 4.00(62.3%); 5.00(13.2%)	4.00(Q1)5.00(Q2)5.00(Q3)	4.00(34.9%); 5.00(65.1%)	177.61	<0.001	<0.001	<0.001	<0.001

68 keV 50%ASIR-V: 68 keV 50% adaptive statistical iterative reconstruction-V, 0.625 mm; 68 keV DLIR-H: 68 keV Deep learning image reconstruction with  high setting (DLIR-H), 0.625 mm; 40 keV DLIR-H: 40keV DLIR-H, 0.625 mm

**Table 4:** Comparison of objective image quality metrics

	Group				Statistical test	
		68 keV 50%ASIR-V	68 keV DLIR-H	40 keV DLIR-H	F value	*P* value
Vessel CT Value		180.20 ± 26.12*	180.79 ± 26.59*	430.97 ± 92.56	333.98	< 0.001
Vessel SD Value		15.11 ± 4.20	12.29 ± 3.41	28.55 ± 7.43	142.11	< 0.001
Liver CT Value		80.10 ± 32.19*	79.66 ± 32.24*	150.21 ± 65.86	40.88	< 0.001
Liver SD Value		17.09 ± 4.13	12.13 ± 3.83	23.65 ± 6.48	71.95	< 0.001
CNR		6.24 ± 1.64	8.43 ± 2.65	11.12 ± 4.29	33.70	< 0.001
ERS		31.45 ± 15.34*	33.10 ± 14.62*	83.73 ± 46.54	53.72	< 0.001

68 keV 50%ASIR-V: 68 keV 50% adaptive statistical iterative reconstruction-V, 0.625 mm; 68 keV DLIR-H: 68 keV Deep learning image reconstruction with high setting (DLIR-H), 0.625 mm; 40 keV DLIR-H: 40 keV DLIR-H, 0.625 mm. * denotes no significant intergroup difference (*p* > 0.05)

## Conclusions

The 40 keV 0.625 mm thin-slice DECT images, when combined with DLIR-H reconstruction, provide superior image quality and high diagnostic confidence for PRETEXT staging of pediatric hepatoblastoma, and may offer potential for future lower-contrast and lower-radiation-dose scanning for this disease.

## Data Availability

The datasets used and/or analyzed during the current study are available from the corresponding author on reasonable request.

## Abbreviations

PRETEXT: Pretreatment Extent of Disease
DECT: Dual Energy CT
DLIR: Deep Learning Image Reconstruction
ASIR-V: Adaptive Statistical Iterative Reconstruction
SD: Standard Deviation
CNR: Contrast-to-Noise Ratio
ERS: Edge Rise Slope
ROI: Regions of Interest
HB: Hepatoblastoma
FBP: Filtered Back Projection
CTA: Computed Tomography Angiography
VMI: Virtual Monoenergetic Imaging
